# Robotic and On-Flow Solid Phase Extraction Coupled with LC-MS/MS for Simultaneous Determination of 16 PPCPs: Real-Time Monitoring of Wastewater Effluent in Korea

**DOI:** 10.3390/toxics13100899

**Published:** 2025-10-20

**Authors:** Sook-Hyun Nam, Homin Kye, Juwon Lee, Eunju Kim, Jae-Wuk Koo, Jeongbeen Park, Yonghyun Shin, Jonggul Lee, Tae-Mun Hwang

**Affiliations:** 1 Korea Institute of Civil Engineering and Building Technology, 283 Goyangdar-Ro, Goyang-Si 411-712, Republic of Korea; fpnsh@kict.re.kr (S.-H.N.); hominkye@kict.re.kr (H.K.); leejw@kict.re.kr (J.L.); kej@kict.re.kr (E.K.); koojaewuk@kict.re.kr (J.-W.K.); jbpark@kict.re.kr (J.P.); shinyonghyun@kict.re.kr (Y.S.); 2 Civil & Environmental Engineering, Korea University of Science & Technology, 217 Gajung-to Yuseong-gu, Daejeon 305-333, Republic of Korea; 3 CentumTech Incorporation, 82 Hwagok-ro 68-gil, Deungchon 1-dong, Seoul 07566, Republic of Korea; jglee@centumtech.co.kr

**Keywords:** advanced real-time monitoring, automation, pharmaceuticals and personal care products, solid-phase extraction, wastewater

## Abstract

Pharmaceuticals and personal care products (PPCPs) are recognized as emerging contaminants of concern, even at ultra-trace concentrations. However, the current detection systems are prohibitively expensive and typically rely on labor-intensive, lab-based workflows that lack automation in sample pretreatment. In this study, we developed a robotic and on-flow solid-phase extraction (ROF-SPE) system, fully integrated with online liquid chromatography-tandem mass spectrometry (LC-MS/MS), for the on-site and real-time monitoring of 16 PPCPs in wastewater effluent. The system automates the entire pretreatment workflow—including sample collection, filtration, pH adjustment, solid-phase extraction, and injection—prior to seamless coupling with LC–MS/MS analysis. The optimized pretreatment parameters (pH 7 and 10, 12 mL wash volume, 9 mL elution volume) were selected for analytical efficiency and cost-effectiveness. Compared with conventional offline SPE methods (~370 min), the total analysis time was reduced to 80 min (78.4% reduction), and parallel automation significantly enhanced the throughput. The system was capable of quantifying target analytes at concentrations as low as 0.1 ng/L. Among the 16 PPCPs monitored at a municipal wastewater treatment plant in South Korea, only sulfamethazine and ranitidine were not detected. Compounds such as iopromide, caffeine, and paraxanthine were detected at high concentrations, and seasonal variation patterns were also observed This study demonstrates the feasibility of a fully automated and on-site SPE pretreatment system for ultra-trace environmental analysis and presents a practical solution for the real-time monitoring of contaminants in remote areas.

## 1. Introduction

Pharmaceuticals and personal care products (PPCPs) encompass a wide range of chemical substances including medications, personal hygiene products, fragrances, disinfectants, and hormones. Due to their persistence, potential for bioaccumulation, and toxicity, PPCPs are recognized as emerging contaminants that can exert physiological effects on both humans and aquatic organisms, even at trace concentrations [1,2,3,4]. PPCPs are considered a major threat to aquatic ecosystems due to their biological activity and environmental persistence, and global monitoring efforts are increasing accordingly [5].

The European Union and the United States Environmental Protection Agency (EPA) have designated 33 priority substances to regulate key organic pollutants responsible for aquatic contamination. Additionally, compounds such as diclofenac, iopamidol, synthetic musks, carbamazepine, ibuprofen, clofibric acid, triclosan, phthalates, and bisphenol A have been proposed as candidates for future regulation [6,7].

To support future regulatory decisions, the European Commission has also implemented a watch list system since 2015 for the systematic monitoring of emerging contaminants in wastewater. The latest update, Implementing Decision 2025/439, was established under Directive 2008/105/EC to guide EU-wide surveillance efforts [European Commission]. Similar monitoring initiatives are being adopted globally in response to increasing concerns over PPCP contamination [8].

Carbamazepine and sulfamethoxazole have been identified as posing high ecological risks to freshwater ecosystems in Asia [2]. Ibuprofen has been shown to inhibit algal growth, disrupt photosynthesis, cause morphological changes in algal cells, and exert immunosuppressive and nephrotoxic effects in fish including alterations in gene expression related to bone development and immune function [9]. The presence of caffeine in aquatic environments has raised concerns as it has been reported to affect the cardiovascular, behavioral, and reproductive systems of both humans and aquatic organisms [10]. Triclosan exhibits chemical properties that can disrupt the endocrine system [11]. Trimethoprim and sulfamethoxazole detected in WWTP effluent were found to enhance antibiotic resistance in two natural bacterial strains present in the receiving environment [6]. Diclofenac is highly toxic and causes damage to the liver, kidneys, and gills of fish such as rainbow trout [12]. Naproxen can affect organisms inhabiting ecosystems either through its inherent toxicity or via the toxicity of its metabolites. The latter can be formed through both physicochemical and biological processes [13]. Paraxanthine is the primary metabolite of caffeine, accounting for more than 80% of its total metabolism [14].

These substances originate from pharmaceutical manufacturing facilities, hospitals, and households and often enter the environment through the improper disposal of unused medications. PPCPs have been widely detected in various environmental matrices including wastewater and sewage [15,16], surface water [17,18,19,20], groundwater, and even drinking water at trace concentrations in the ng/L range [21,22,23,24]. Frequently detected PPCPs in the United States include caffeine, ibuprofen, acetaminophen, triclosan, carbamazepine, and various antibiotics [19,25]. Ibuprofen is commonly reported [26], while in Mexico, diclofenac, ibuprofen, naproxen, and clofibric acid have been identified [27]. In China, roxithromycin, erythromycin, ibuprofen, carbamazepine, propranolol, triclosan, and antibiotics are frequently found [28,29,30,31], and in South Korea, commonly detected PPCPs include acetaminophen, ibuprofen, caffeine, carbamazepine, naproxen, and antibiotics [32,33].

PPCPs are of particular concern because conventional wastewater treatment plants often show insufficient removal efficiencies, allowing both parent compounds and transformation products with similar pharmacological activity to be discharged into aquatic ecosystems [33]. As a result, domestic wastewater is considered a significant source of ecological contamination and potential human health risk [34,35]. Therefore, the accurate and sensitive monitoring of PPCPs in environmental waters is essential.

Recent advances in analytical technologies have enabled the simultaneous quantification of multiple PPCPs at trace levels across different aquatic systems. Automated workflows integrating extraction, separation, and detection—such as online solid-phase extraction (SPE) coupled with liquid chromatography-tandem mass spectrometry (LC-MS/MS)—have become widely adopted for environmental sample analysis due to their advantages of minimal sample preparation, reduced sample volume, and lower solvent consumption [36,37,38,39,40].

In previous studies, online SPE systems typically referred to fully automated analytical workflows in which extraction and quantification were seamlessly integrated with LC-MS/MS instruments in real-time [41,42,43,44,45]. However, most existing systems still require the transport of collected samples to laboratories for further processing and analysis, and often rely on expensive SPE modules and mass spectrometry instruments. To date, no system has been developed that enables fully automated, on-site sampling, sample preparation, injection, and real-time analysis within a single platform.

SPE remains the most critical step in trace-level PPCP analysis, but its automation and field integration have remained technically challenging. This study addresses this gap by developing a robotic, on-flow SPE system designed specifically for autonomous on-site monitoring.

Recently, robotic systems and on-flow technologies have been utilized to automate sample preparation in chromatography and mass spectrometry analyses [46].

Accordingly, this study integrated two major automation approaches—robotic systems and on-flow technologies—to develop a field-deployable robotic on-flow solid-phase extraction (ROF-SPE) system capable of fully automated on-site sampling, concentration, injection, and analysis within a single platform. The system actively controls the flow rate using solenoid valves and precision pumps while integrating multi-step extraction and concentration processes directly with the analytical instrument in an online configuration, thereby maximizing the throughput and significantly reducing the analysis time. The automated sample pretreatment process was tested to verify the accuracy, precision, and reproducibility of the analysis while minimizing human error and exposure to hazardous chemicals. The objective of this study was to overcome the limitations of conventional online SPE systems by developing a fully automated, field-based environmental monitoring technology capable of real-time analysis, ensuring that the robot-controlled on-flow SPE system achieves precision and efficiency equivalent to conventional offline or semi-automated methods while shortening the analysis time and enabling the stable, long-term real-time monitoring of PPCPs on-site.

## 2. Materials and Methods

### 2.1. Chemicals and Reagents

All solvents used here were of LC-MS grade, whereas all other chemicals were of analytical reagent grade. Methanol (≥99.9%, suitable for HPLC) was sourced from Avantor (Radnor, PA, USA), and formic acid (96%, ACS grade) and ammonium hydroxide (28–30%, ACS grade) were obtained from Sigma-Aldrich (St. Louis, MO, USA). The 16 pharmaceutical regents of acetaminophen (ACP), atenolol (ATN), ranitidine (RNT), paraxanthine (PRX), iopromide (IPM), caffeine (CFI), sulfamethazine (SFA), trimethoprim (TMP), sulfamethoxazole (SFX), lincomycin (LCM), propranolol (PPN), carbamazepine (CBZ), naproxen (NPX), diclofenac (DCF), ibuprofen (IBF), and triclosan (TCS) were purchased from Sigma-Aldrich (St. Louis, MO, USA).

Internal standard substances—used to control the variability of the target analytes and eliminate matrix effects and instrumental interferences to improve quantification accuracy and reproducibility—included ACP-d_4_, ATN-d_7_, RNT-d_6_, PRX-d_3_, IPM-d_3_, CFI-d_9_, TMP-d_9_, SFX-d_4_, LCM-d_3_, PPN-d_7_, CBZ-d_10_, NPX-d_4_, DCF-d_4_, IBF-d_3_, TCS-d_3_, and SFA-^13^C_6_. The standards were purchased from Sigma-Aldrich (St. Louis, MO, USA). To evaluate the SPE recovery, seven internal standards were employed: CBZ-d_10_, IBF-d_3_, ATN-d_7_, TMP-d_9_, SFA-^13^C_6_, SFX-d_4_, and DCF-d_4_. Stock solutions of all of the compounds were prepared in methanol at a concentration of 1000 mg/L. Deionized water used for the preparation and dilution of standard solutions was produced using a purification system with a resistivity of 18.2 MΩ·cm at 25 °C. Table 1 lists the key physicochemical properties of the 16 PPCPs investigated.

### 2.2. The Robotic and On-Flow SPE System

**Figure 1:**
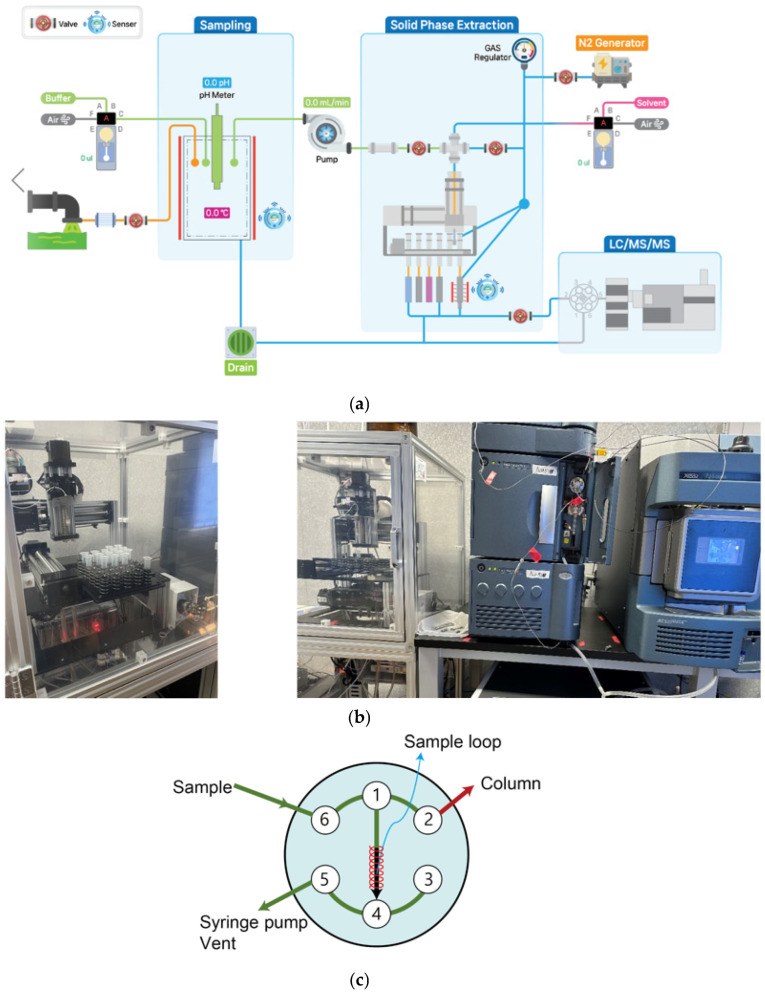
Schematic diagram of the robotic and on-flow SPE coupled with liquid chromatography-tandem mass spectrometry; (**a**) process flowchart, (**b**) SPE conceptual design and fabricated SPE device, and (**c**) 2-position, 6-port valve.

We developed a robotic and on-flow SPE system coupled with a liquid chromatography-tandem mass spectrometry (ROF-SPE-LC-MS/MS) system capable of performing sample collection and SPE pretreatment directly in the field. A conceptual diagram of the system is shown in Figure 1a. The “solid phase extraction” and “LC/MS/MS” sections in Figure 1a correspond to the SPE components and the overall system shown in Figure 1b. Furthermore, the flow of valves depicted in the “solid phase extraction” section of Figure 1a is illustrated in greater detail in Figure 1c.

The ROF-SPE system, along with a liquid chromatography (LC) system (Acquity, Waters, Milford, MA, USA) and a tandem mass spectrometer (Xevo TQ-S, Waters, Milford, MA, USA), was modularly installed and integrated within a 20-foot container (2.3 m wide × 6.0 m long × 2.3 m high; load capacity: 25 metric tons). The container was positioned near the tertiary effluent outlet of the I City wastewater treatment plant and designed to allow mobile deployment. At the site, tertiary effluent was directly sampled using a pump, automatically filtered through a 0.5 μm filter (SACAR, Seoul, Republic of Korea). A pH meter (HI-1230B, HANNA, Seoul, Republic of Korea) and a temperature sensor (GA100S, Samwonen, Republic of Korea) were installed in the sampling unit to adjust the pH and monitor the temperature of the sample before it was introduced into the ROF-SPE system.

In the field, samples automatically extracted through the SPE process were loaded and discharged from a sample loop using a 2-position, 6-port valve (Rheodyne, CA, USA). Subsequently, the samples were automatically injected into the LC-MS/MS system via a column. The SPE component was designed to accommodate 24 SPE cartridges mounted on a linear-motion tray, allowing for sequential use (Figure 1b). In the 2-position, 6-port valve shown in Figure 1c, the pre-concentrated sample from the SPE system is loaded into the loop (ports 1–4) via the syringe pump (port 5) through port 6. After filling, the valve switches from the loading path (LC pump → 3 → 2 → column) to the injection path (3 → 4 → loop → 1 → 2 → column) to deliver the sample to the column.

The concentration process is carried out through vortex shearing and heating of a custom-made sample block (CentumTech, Seoul, Republic of Korea) at 40 °C and 30 psi. The vortex shearing caused by the gas nozzle (2 mm, CentumTech, Seoul, Republic of Korea) is a process in which nitrogen gas flows along the inner wall, inducing rotation of the sample. This rotation increases the surface area of the sample, thereby maximizing its contact with the concentrating gas. Nitrogen gas, known for its low oxidative properties, is used in this process. Once concentration is complete, a sensor (HPF-T032E-L02, Azbil, Tokyo, Japan) mounted at the bottom measures the sample status. This value is then converted into an on/off signal via the sensor amplifier (HPX-EG00-1S-L02, Azbil, Tokyo, Japan). When the preset value is reached, the concentration process automatically stops.

The concentrated sample obtained through the SPE pretreatment was automatically introduced into the LC-MS/MS injection port using a 3-port syringe pump (IMI Norgren, Littleton, CO, USA) and sensor (Azbil, Tokyo, Japan).

The ROF-SPE system was designed to be compatible with various LC-MS/MS platforms. The LC-MS/MS interface supports multiple communication protocols including TTL, RS232C, and RS485, and incorporates a timeline-based mode that allows for start and end signal control from either the LC pump or the mass spectrometer.

### 2.3. Liquid Chromatography-Tandem Mass Spectrometry

Chromatographic separation was performed using a Waters BEH C18 column (2.1 × 100 mm, 1.9 μm) maintained at 45 °C. The mobile phase consisted of 0.1% formic acid in water (A) and methanol (B). A gradient elution was performed as follows: 95% B at 0 min, decreased to 80% at 1 min, further to 0% at 10 min, held for 12 min, and returned to 95% at 15 min. The flow rate was set at 0.3 mL/min, and the injection volume was 10 μL. Mass spectrometry detection was performed using an electrospray ionization source in both positive and negative ion modes. The nebulizer gas (ion source 1) and heater gas (ion source 2) were supplied at 50 psi, while the curtain gas was maintained at 25 psi. The desolvation temperature was set to 500 °C. Ion spray voltages were +5.5 and −4.5 kV for the positive and negative modes, respectively. Nitrogen was used as the collision gas throughout the analysis.

### 2.4. The Robotic and On-Flow Solid-Phase Extraction Method

The robotic and on-flow solid-phase extraction (ROF-SPE) was fully automated. A 500 mL sample of final effluent from a sewage treatment plant was automatically filtered through a 0.5 μm filter. To suppress pharmaceutical degradation caused by metal-catalyzed reactions, 100 μL of Na_2_EDTA was added via syringe pump injection [47,48]. Seven internal standards were automatically introduced to a final concentration of 100 µg/L to evaluate extraction recovery. The pH was adjusted to the desired condition using 0.1% (*v*/*v*) formic acid and 0.1% (*v*/*v*) ammonium hydroxide.

The SPE cartridge used was a commercially available Oasis HLB (200 mg, 6 cc, 30 µm; Waters Corporation, Milford, MA, USA), which provides high retention for acidic, neutral, and basic compounds and is suitable for water-based sample loading [44,49,50]. Cartridges were preconditioned with 6 mL of methanol (3 mL/min), followed by 6 mL of water (3 mL/min), and equilibrated with 6 mL of water at pH 3, 7, or 10 (5 mL/min) depending on the experimental conditions.

The sample (500 mL) was then loaded onto the cartridge at 10 mL/min. Washing and eluent are important to increase the recovery rates and remove matrix interferences [51]. Cartridges were washed with 6–18 mL of water at pH 3, 7, or 10 (3 mL/min), adjusted using 0.1% formic acid or 0.1% ammonium hydroxide. Washing was compared using an organic solvent (10% MeOH) at pH 3, 7, and 10.

The cartridge was subsequently dried under nitrogen gas for 20 min to remove residual moisture. Elution was performed in two steps using 3–6 mL of methanol (1 mL/min). The eluate was evaporated under nitrogen at 40 °C and 30 psi for 20 min, then reconstituted in 1 mL of methanol for LC-MS/MS analysis. The total pretreatment time of the FAOS-SPE system was 150 min for the first sample. When operated continuously, the system was capable of processing up to 18 samples per day (80 min).

Recovery tests were conducted in five replicates, and the average recovery was used for evaluation. The developed ROF-SPE system was compared with a commercial offline SPE device (AquaTrace ASPE899, GLtechno Holdings Inc., Tokyo, Japan) under identical pretreatment conditions.

Under consistent pretreatment conditions (flow rate, solvent volume, sample volume, processing speed, drying, and elution parameters), the offline SPE method required 220 min for evaporation alone. The total pretreatment time per sample was 370 min.

### 2.5. Method Validation

Method detection limits (MDLs) and method quantification limits (MQLs) were experimentally determined based on signal-to-noise (S/N) ratios of 3 and 10, respectively. The MDL and MQL were verified by injecting the standard solutions five times at the target concentration level. Calibration curves were established by linear regression using six different concentrations. Precision was also evaluated using five replicate extractions and expressed as the relative standard deviation (RSD, %) of the repeated measurements. The accuracy was determined as the relative error (%) between the measured and spiked concentrations.

### 2.6. Matrix Effect and SPE Extraction Recovery

Matrix effects (ME, %) were evaluated using the sample as the analytical matrix. The ME was determined by comparing the average peak areas of the analytes spiked into the samples (*A_spike_*) with those of standards prepared in solvent (*A_solvent_*) after subtracting the background response from the unspiked sample (*A_blank_*). Each condition was analyzed in triplicate, and the ME calculated according to the following equation [41,52]:

(1) $$ME\left(\%\right)=\frac{A_{spike}-A_{blank}}{A\;solvent}\times 100$$

Here, ME values within the range of 85–115% were considered acceptable without correction. Values < 85% indicated ion suppression, while those > 115% indicated ion enhancement, both of which require correction using internal standards [53]. The RSD should not exceed 15% [54]. Extraction recovery was determined by comparing the concentration obtained after SPE pretreatment with the internal standard and the initial addition concentration, and was estimated as the average of the five experiments [41].

(2) $$Extraction\;recovery\left(\%\right)=\frac{C_{spike}-C_{blank}}{C_{actual}}\times 100$$

The process efficiency was calculated as the overall efficiency reflecting both the ME and recovery [52].

### 2.7. Water Quality

The pH and total dissolved solids (TDS) were measured using a multi-parameter meter (ORION STAR A221, Thermo Scientific, Waltham, MA, USA). Turbidity was determined using a turbidimeter (2100N, HACH, Loveland, CO, USA), and the total organic carbon (TOC) was analyzed using a TOC analyzer (TOC-VCPH, Shimadzu, Kyoto, Japan). The UV254 absorbance was measured using a UV–Vis spectrophotometer (DR5000, HACH, Loveland, CO, USA). Total nitrogen (T-N) and total phosphorus (T-P) were determined using a multi-parameter photometer (SYNCA 3ch; BLTech Korea, Chuncheon-si, Republic of Korea).

## 3. Results

### 3.1. LC-MS/MS Optimization

A field-deployable ROF-SPE coupled with the LC-MS/MS method was developed to analyze PPCPs in tertiary effluent from a wastewater treatment plant. The presence of various water constituents, such as specific enzymes, fulvic and humic acids, and microorganisms, can lead to the formation of diverse metabolic products that markedly affect PPCP recovery. Matrix effects can suppress or, in rare cases, enhance the analytical signals of target compounds, occasionally resulting in inaccurate results [55]. PPCPs are mostly polar or moderately polar compounds [56,57]. Therefore, the accurate quantification of different types of water requires the careful consideration of all water quality characteristics that may influence analyte recovery. Table 2 presents the water quality parameters of tertiary effluent at the study site. The MS conditions were optimized based on previously reported ion fragments, and the final parameters are summarized in Table 3. Isotopically labeled compounds were used as internal standards for calibration curve construction and quantification of the target analytes, facilitating matrix effect correction and improving analytical precision.

### 3.2. Method Validation

The performance of the developed method was evaluated in terms of its MDL, MQL, precision, and accuracy, as summarized in Table 4. All 16 target PPCPs demonstrated excellent linearity across the tested concentration range (*n* = 6) with correlation coefficients (R^2^) > 0.99. The MDLs and MQLs ranged from 2.5 to 103.4 ng/L and 3.6 to 328.9 ng/L, respectively. Among the analytes, PRX exhibited notably higher MDL and MQL than those of the other 15 compounds. The RSD for precision ranged from 3.0 to 15.4%, whereas accuracy values ranged from 80.6 to 104.9%. These results confirm that the method satisfied the standard analytical validation criteria with acceptable precision (RSD < 25%) and accuracy within the range of 85–110%.

### 3.3. Matrix Effect

To account for potential matrix interference, the ME was evaluated by spiking the 16 target PPCP standards into real field samples and assessed the extent of ion suppression or enhancement as well as the sensitivity of each compound to MEs. Three concentration levels (1, 5, and 20 μg/L) were tested. The ME results for each compound are listed in Table 5. At concentrations of 1 and 5 μg/L, notable ion suppression (ME < 85%) was observed for PRX (73.2%), SFX (78.9%), IBF (75.6%), and TCS (61.9%). In contrast, signal enhancement (ME > 115%) was observed for PPN and DCF, with ME values of 136.5 and 120.7%, respectively. Greater suppression was observed at 1 μg/L compared with 5 μg/L for most compounds. At 20 μg/L, all compounds showed ME values ranging from 80.4 to 117.8%, falling within the generally accepted range of 85–115%. The RSD of all measurements remained <15% for all tested concentrations. For compounds affected by matrix-related ion interference, calibration was performed using isotopically labeled surrogates to compensate for signal variability.

### 3.4. Optimization of Robotic and On-Flow SPE Method

The development of a pretreatment method capable of detecting PPCPs across diverse chemical classes remains a considerable challenge. Therefore, the pretreatment procedure was optimized specifically for 16 target PPCPs, prioritized based on their anticipated occurrence in the environment.

The results applied to the FAOS-SPE system were applied to the offline SPE system and compared. SPE was performed using 500 mL of sample, which had been filtered on-site through a 0.5 μm filter, and concentrated onto an Oasis HLB cartridge to achieve a concentration factor of 500. The 16 target PPCPs were presumed to be weakly basic or acidic, exhibiting a range of physicochemical properties.

The sample pH was adjusted to 3 (using 0.1% (*v*/*v*) formic acid), 7 (using deionized water), and 10 (using 0.1% (*v*/*v*) ammonium hydroxide), and the recovery rates at each pH were compared to evaluate the effect of pH on extraction efficiency. To minimize the loss of target analytes during SPE pretreatment and maximize recovery, it is essential to control not only the sample pH, but also the pH of the loading and washing solvents.

**Figure 2:**
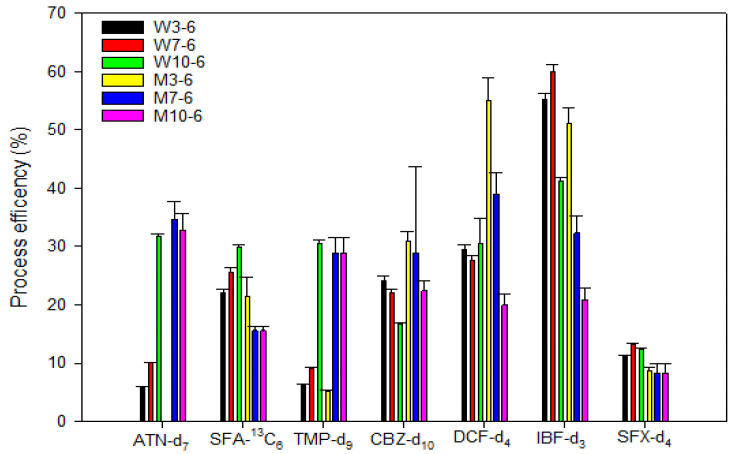
Process efficiency (%) of seven isotopically labeled compounds under six washing conditions using water and 10% methanol at pH 3, 7, and 10.

Figure 2 presents the process efficiency (%) of seven isotopically labeled compounds: ATN-d_7_, SFA-^13^C_6_, TMP-d_9_, CBZ-d_10_, DCF-d_4_, IBF-d_3_, and SFX-d_4_, evaluated under six different washing conditions. These conditions included 6 mL of water adjusted to pH 3, 7, and 10 (W3-6, W7-6, and W10-6), and 6 mL of 10% methanol adjusted to the same pH values (M3-6, M7-6, and M10-6). Under the washing conditions using water at pH 3, 7, and 10, most compounds showed the highest efficiency at pH 7. In particular, IBF-d_3_ exhibited a very high recovery rate (over 60.9%) under pH 7. When washed with 10% MeOH, the efficiency generally decreased compared with water washing. Only DCF-d_4_ showed a high recovery (58.9%) under the pH 3 condition with 10% MeOH. For TMP-d_9_ and SFX-d_4_, the efficiency did not change significantly, even under the 10% MeOH washing conditions. The addition of 10% MeOH led to reduced efficiency in some compounds, and washing with 10% MeOH at pH 10 tended to show the lowest efficiency, indicating it was the least effective condition.

The washing solvent was adjusted to match the sample pH conditions using water with pH values of 3, 7, and 10. The sample pH values were 3, 7, and 10. The water washing volumes (WWV) were varied between 6, 12, and 18 mL, while the elution volumes were adjusted to 6, 9, and 12 mL to evaluate their effects on analyte recovery.

**Figure 3:**
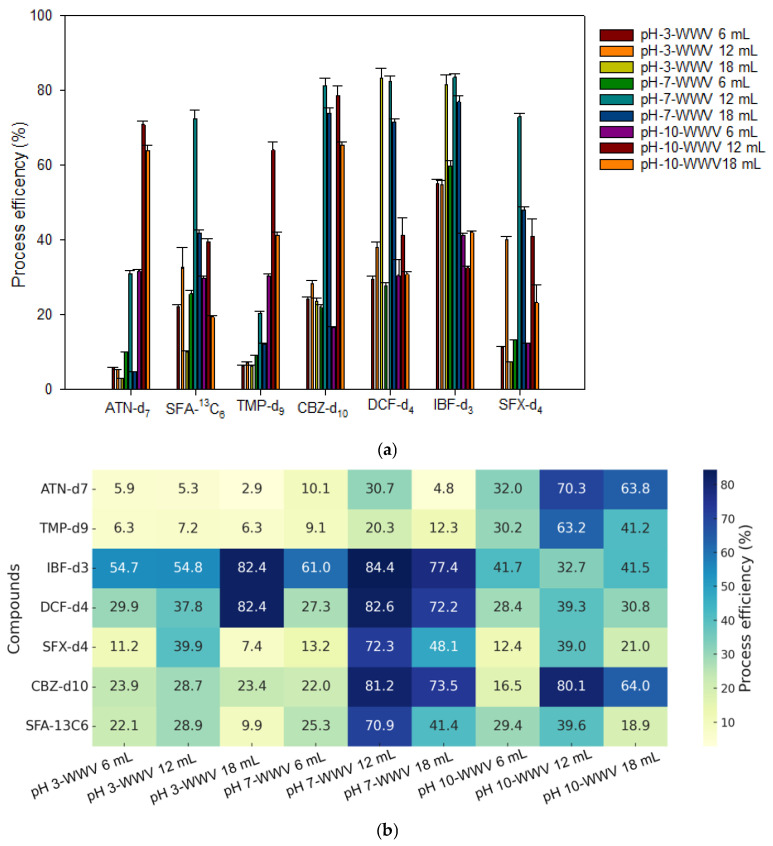
Results of extraction using the internal standard: (**a**) Process efficiency (PE) results under the elution 6 mL condition, varying by pH (3, 7, and 10) and water washing volumes (6, 12, and 18 mL). (**b**) Heatmap visualization to identify optimal SPE conditions based on compound-specific PE.

For the SPE recovery test, internal standards were selected based on the pKa values of the target compounds listed in Table 1. Specifically, samples were spiked with internal standards under the following pH conditions: pH 3 for acidic compounds (IBF-d_3_, DCF-d_4_, and SFA-^13^C_6_), pH 7 for neutral compounds (CBZ-d10 and SFX-d4), and pH 10 for basic compounds (ATN-d_7_ and TMP-d_9_). Extraction recovery was calculated based on spiked samples, and the final process efficiency was determined by considering the ME. The extraction results (Figure 3a) show the process efficiency under an elution volume of 6 mL across the three sample pH conditions (pH 3, 7, and 10) and three washing volumes (6, 12, and 18 mL). Figure 3b shows the heatmap visualization used to determine the optimal extraction conditions.

Under pH 3 conditions, ATN-d_7_, TMP-d_9_, and SFX-d_4_ showed poor recoveries (<11%), while DCF-d_4_ and IBF-d_3_ exhibited relatively higher recoveries (>80%). However, the process efficiency values for most compounds at pH 3 were generally low, indicating that pH 3 is an inefficient condition. The highest overall recoveries were observed at pH 7, particularly with a washing volume of 12 mL, where compounds such as CBZ-d_10_, IBF-d_3_, DCF-d_4_, SFX-d_4_, and SFA-^13^C_6_ showed process efficiency values ranging from 70 to 84%. Under pH 10, basic compounds like ATN-d_7_ and TMP-d_9_ exhibited increased process efficiency values, reaching up to 70.3%. However, most other compounds showed lower recoveries (12.4–41.7%), indicating that this condition is not suitable for all analytes. Among all of the tested conditions, a washing volume of 12 mL consistently yielded the most stable and highest recoveries and is therefore recommended as the optimal washing condition for the ROF-SPE procedure.

**Figure 4:**
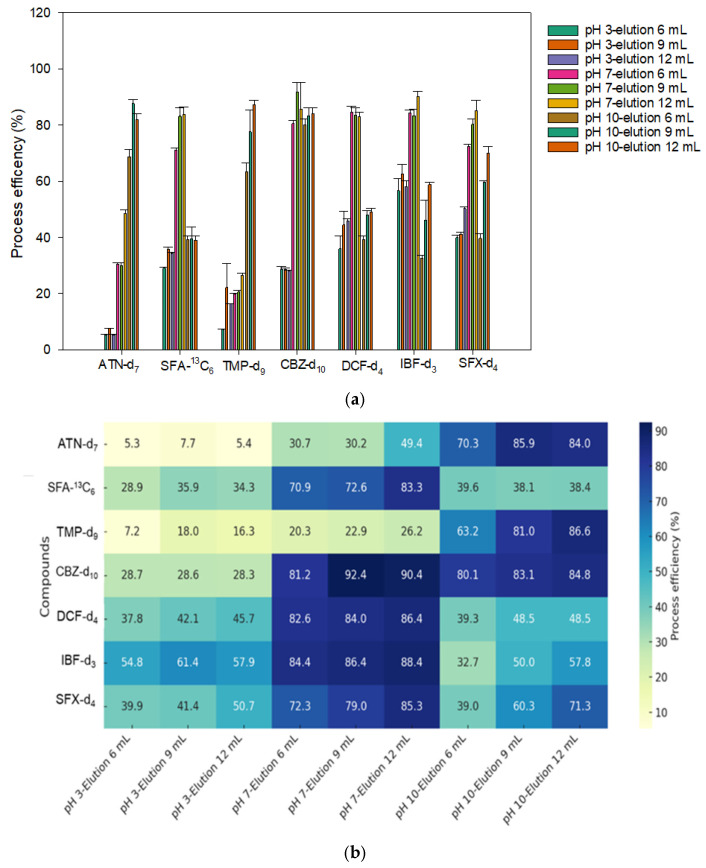
Optimization results of extraction using internal standard. (**a**) Process efficiency (PE) results under the washing 12 mL condition, varying by pH (3, 7, and 10) and elution volumes (6, 9, and 12 mL). (**b**) Heatmap visualization to identify optimal SPE conditions based on compound-specific PE.

Under an optimized washing volume of 12 mL, the elution volume was varied (6, 9, and 12 mL) and tested at pH 3, 7, and 10. The resulting process efficiency data are presented in Figure 4a. At pH 3, the process efficiency values were generally low, ranging from 5.3 to 61.4%. The highest recoveries were observed at an elution volume of 9 mL for IBF-d_3_ (61.4%) and SFX-d_4_ (41.4%). Increasing the elution volume beyond 9 mL resulted in only a limited improvement in recovery. At pH 7—with the exception of ATN-d_7_ and TMP-d_9_—the process efficiency values ranged from 70.9 to 92.4% with the highest recoveries observed at an elution volume of 12 mL. However, acceptable recoveries were also obtained at 9 mL elution for several compounds including CBZ-d_10_ (92.4%), DCF-d_4_ (84.0%), IBF-d_3_ (84.6%), SFX-d_4_ (79.0%), and SFA-^13^C_6_ (82.9%). These results indicate that even acidic compounds can achieve sufficient recovery under neutral pH conditions when combined with optimal washing and elution settings.

At pH 10, an elution volume of 9 mL yielded high recoveries for basic compounds such as ATN-d_7_ (85.9%) and TMP-d_9_ (80.9%) as well as CBZ-d_10_ (83.1%), suggesting that this condition is favorable for the pretreatment of basic analytes. With the exception of ATN-d_7_ and SFA-^13^C_6_, the process efficiency values obtained with an elution volume of 12 mL were only marginally higher (<2–6%) than those observed with 9 mL (Figure 4b). Considering that such a minor improvement (2–3%) would require additional solvent consumption, which is inefficient from both an economic and environmental perspective, 9 mL was deemed sufficient for elution.

Most target PPCPs, including acidic and neutral compounds, consistently exhibited high and stable process efficiency values at pH 7, whereas some basic compounds such as ATN-d_7_ and TMP-d_9_ showed improved recoveries at pH 10. Therefore, the optimal extraction conditions were selected by combining the 9 mL elution volumes at pH 7 and 9, offering a robust and efficient compromise across all classes of analytes. The process efficiency tests verified the quality, consistency, and reliability of the SPE procedure.

A comparative analysis of offline SPE and optimized ROF-SPE conditions was conducted using five replicates. The RSDs of the seven analytes used for process efficiency testing were all within 15% for both methods. The recoveries of all target compounds ranged from 80.7 to 119.9%. The intra- and inter-day precision values, expressed as RSDs, were within 10.6 and 15.6%, respectively.

These satisfactory recovery and precision results demonstrate that the developed online analytical method provides excellent accuracy and reproducibility comparable to those of conventional offline SPE systems.

### 3.5. Analysis of Real Water Samples

**Figure 5:**
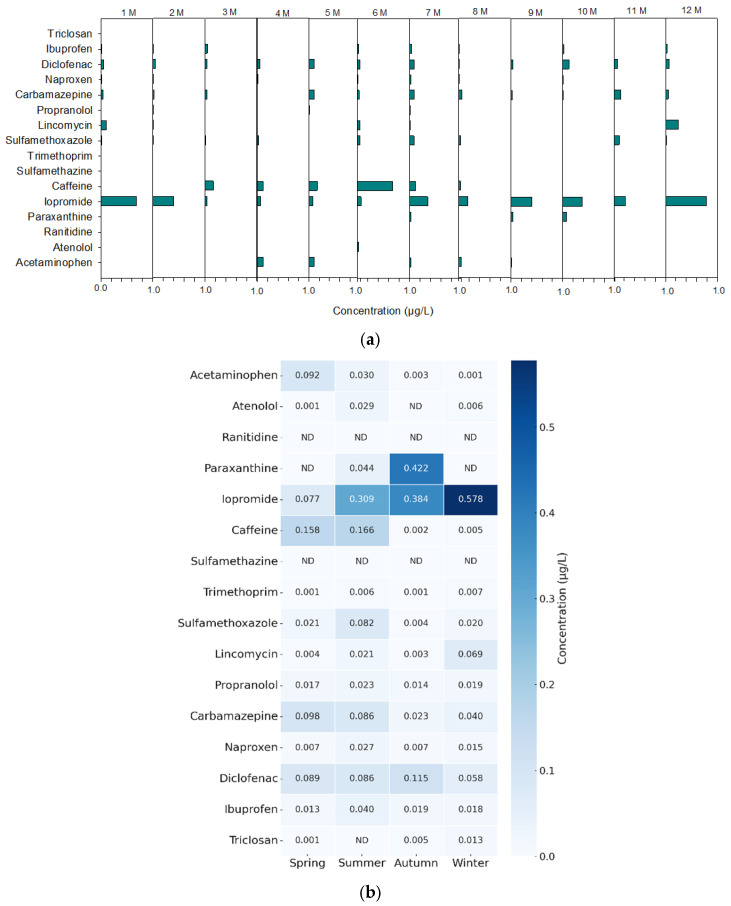
Concentrations of 16 target PPCPs in wastewater effluent analyzed using the robot and of-flow SPE–LC–MS/MS system: (**a**) variation in concentrations across the monitoring period; (**b**) seasonal variation. “ND” indicates not detected.

The proposed optimized ROF-SPE extraction method, which involves alternating operation at pH 7 and 10 with a 12 mL water washing step and 9 mL elution, was successfully applied and implemented for on-site monitoring of the tertiary effluent from a wastewater treatment plant. Monitoring results obtained from September 2024 to July 2025 are presented in Figure 5. Figure 5a illustrates the monthly distribution of 16 PPCPs detected in the tertiary effluent from January to December. Most compounds were detected at trace levels (<1.0 µg/L), with intermittent appearances depending on the month.

The detected concentration ranges (μg/L) for the 16 target PPCPs were as follows: ACP (ND–0.1353), ATN (ND–0.0337), PRX (ND–0.5236), IPM (0.0118–0.7914), CFI (ND–0.6870), TMP (ND–0.0068), SFX (ND–0.1094), LCM (ND–0.2524), PPN (ND–0.0406), CBZ (ND–0.1421), NPX (0.0002–0.1325), DCF (0.0188–0.1999), IBF (0.0006–0.1601), and TCS (ND–0.0182). IPM and CFI showed consistent detections throughout the year, with noticeably higher concentrations during the summer months (June to August), while PRX exhibited peaks in early and late periods of the monitoring. Relatively high levels of DCF, LCM, IBF CBZ, and NPX were also detected. SFX, ACP, PPN, and ATN were detected in trace amounts, whereas SFA and RNT were rarely detected.

The seasonal average concentrations were grouped into spring (March–May), summer (June–August), autumn (September–November), and winter (December–February) (Figure 5b). It was operated for more than 30 days in each season. Among the analytes, CFI, PRX, and IPM exhibited relatively higher average concentrations during summer and autumn, with IPM also showing elevated levels in winter.

These seasonal variations likely reflect changes in usage patterns and environmental factors such as rainfall and temperature. This highlights the importance of considering seasonal variability when developing pollutant management strategies because the occurrence and persistence of PPCPs may vary across seasons. Such fluctuations can be effectively monitored using the ROF-SPE-LC-MS/MS system, which enables a rapid and continuous field-based analysis. Compared with conventional offline SPE—which requires ~370 min to run including sample pretreatment—the ROF-SPE system reduced the total processing time to 130 min, resulting in a time reduction of nearly 65%. In addition, during LC-MS/MS analysis, the next sample can be prepared in parallel, allowing for a continuous and efficient analytical workflow.

The ROF-SPE-LC-MS/MS method offers notably greater convenience and efficiency than traditional offline SPE methods, with the added advantage of on-site applicability. The proposed method enabled the ultra-trace quantification (as low as 0.1 ng/L) of 16 PPCPs commonly found in wastewater effluent, making it a valuable tool for effective environmental monitoring.

## 4. Discussion

This study successfully demonstrated the development and field deployment of a robotic and on-flow solid-phase extraction (ROF-SPE) system coupled with LC–MS/MS for the real-time monitoring of pharmaceuticals and personal care products (PPCPs) in wastewater effluent. The system performed reliably for 12 months under real-world conditions, validating its analytical precision, recovery, and operational stability for continuous field applications.

Conventional offline SPE–LC–MS/MS methods generally require labor-intensive sample preparation, extended analysis time, and chemical preservation steps before laboratory analysis. In contrast, the developed ROF-SPE system automates all processes including sample collection and injection on-site, reducing the total analysis time from approximately 370 min to 80 min (a 78.4% reduction) while maintaining comparable precision (RSD ≤ 15%). Conventional online SPE or passive sampling approaches, such as osmotic pump SPE [58], have been limited to partial automation or long-term accumulation methods, making real-time monitoring impossible and requiring chemical preservation or off-site processing [59,60]. The present system thus provides a distinct advancement by enabling real-time, robotic, on-site sample injection, and direct LC–MS/MS integration, representing a fully autonomous field monitoring solution.

The optimized ROF-SPE method achieved high recovery efficiencies of 72.6–92.4% for most compounds and up to 85.9% for basic analytes such as atenolol and trimethoprim. These results are consistent with those reported in previous studies using advanced systems such as conventional online or automated SPE–LC–MS/MS methods [61,62].

Furthermore, the system achieved a limit of quantification as low as 0.1 ng/L, which aligns with or surpasses the detection capabilities of previously published PPCP monitoring methods in wastewater [62]. This performance demonstrates that full automation and field integration do not compromise analytical sensitivity or reproducibility.

Among the 16 target PPCPs, 14 were consistently detected across seasons including IPM, CFI, PRX, LCM, DCF, IBF, and CBZ. The compounds detected in this study have also been reported at high levels in previous studies, suggesting that the PPCPs identified in domestic effluents are similar to those detected internationally [22,63]. The PPCPs exhibited seasonal variations [64], which are likely attributed to increased pharmaceutical consumption and changes in hydraulic load [63,65].

The complete automation of the pretreatment workflow, which covers sample collection, filtration, pH adjustment, extraction, and injection, marks a significant advancement in field-deployable environmental analysis. This system reduces human error, lowers solvent consumption, and eliminates the need for chemical preservatives, thereby promoting greener analytical practices.

Nonetheless, potential limitations remain including matrix interferences caused by variations in wastewater composition and the requirement for periodic calibration during extended use. Future research should aim to expand the range of target analytes including endocrine-disrupting compounds, pesticides, and both perfluoroalkyl and polyfluoroalkyl substances. Overall, the developed ROF-SPE–LC–MS/MS platform provides a practical and scalable solution for the autonomous monitoring of PPCPs in wastewater. Compared with previously reported online or automated systems, it offers faster analysis, comparable or improved recoveries, and reliable field applicability [40,41]. These results demonstrate that robotic and on-flow integration can bridge the gap between laboratory precision and real-world environmental surveillance, advancing the transition toward fully automated, data-driven water quality monitoring systems.

## 5. Conclusions

This study developed a fully automated robotic on-flow solid-phase extraction (ROF-SPE) coupled with LC-MS/MS for the real-time, on-site monitoring of pharmaceuticals and personal care products (PPCPs) in wastewater effluent. The system integrates all stages of sample pretreatment, including collection, filtration, concentration, injection, and analysis, into a single automated platform. This approach eliminates manual handling and enables continuous and autonomous operation in the field.

Optimization of the pretreatment conditions showed that applying 12 mL water washing and 9 mL elution at pH 7 resulted in recoveries of 72.6–92.4% for most target compounds (e.g., IBF-d_3_, DCF-d_4_, CBZ-d_10_, SFX-d_4_, SFA-^13^C_6_) with excellent reproducibility (RSD ≤ 15%). Under pH 10 conditions, basic compounds such as ATN-d_7_ and TMP-d_9_ achieved recoveries of up to 81.0%, confirming stable extraction performance across acidic, neutral, and basic analytes.

Compared with the conventional offline SPE method, the developed system maintained equivalent precision (RSD ≤ 15%) while reducing the most time-consuming evaporation step to only 20 min. As a result, the total analysis time decreased from approximately 370 min to 80 min (a 78.4% reduction). Moreover, parallel sample preparation during LC–MS/MS analysis allowed up to 18 analyses per day, significantly improving the analytical throughput. This system was capable of quantifying target compounds at concentrations as low as 0.1 ng/L. On-site operation of the ROF-SPE–LC-MS/MS system at a municipal wastewater treatment plant from September 2024 to July 2025 demonstrated its operational stability under real conditions. Among the 16 targeted PPCPs, iopromide (0.0118–0.7914 µg/L), caffeine (≤0.6870 µg/L), and paraxanthine (≤0.5236 µg/L) were detected at the highest concentrations, followed by lincomycin, diclofenac, ibuprofen, and carbamazepine. Seasonal variations revealed higher average concentrations during summer and autumn, likely due to differences in usage patterns and environmental factors such as rainfall and temperature.

These findings demonstrate that the proposed ROF-SPE–LC-MS/MS system offers high accuracy, reproducibility, and robustness for long-term field operation. The technology represents a major step forward in autonomous environmental monitoring and has strong potential for continuous water quality assessment, pollution response, and early warning applications.

## Figures and Tables

**Table 1:** Physico-chemical properties of the 16 target pharmaceuticals and personal care products.

Compounds	Abbreviation	pKa	Log K_ow_	Pv (mmHg)	MW	Molecular Formula
Acetaminophen	ACP	9.38	0.46	7 × 10^−6^	151.17	C_8_H_9_NO_2_
Atenolol	ATN	9.6	0.16	2.924 × 10^−10^	266.34	C_14_H_22_N_2_O_3_
Ranitidine	RNT	8.2	0.27	1.2 × 10^−7^	314.41	C_13_H_22_N_4_O_3_S
Paraxanthine	PRX	0.9	−0.07	7.6 × 10^−5^	180.16	C_7_H_8_N_4_O_2_
Iopromide	IPM	8.4	−3.1	1.4 × 10^−20^	791.1	C_18_H_24_I_3_N_3_O_8_
Caffeine	CFI	14	−0.07	2.5 × 10^−3^	194.19	C_8_H_10_N_4_O_2_
Sulfamethazine	SFA	7.4	0.14	5.2 × 10^−8^	264.30	C_11_H_12_N_4_O_2_S
Trimethoprim	TMP	7.12	0.91	9.88 × 10^−9^	290.32	C_14_H_18_N_4_O_3_
Sulfamethoxazole	SFX	6.0	0.89	6.93 × 10^−8^	253.28	C_10_H_11_N_3_O_3_S
Lincomycin	LCM	7.6	0.29	2.7 × 10^−20^	406.54	C_18_H_34_N_2_O_6_S
Propranolol	PPN	9.5	3.48	1.7 × 10^−5^	259.80	C_16_H_21_NO_2_
Carbamazepine	CBZ	7	2.47	1.84 × 10^−7^	236.27	C_15_H_12_N_2_O
Naproxen	NPX	4.15	3.18	1.892 × 10^−6^	230.27	C_14_H_14_O_3_
Diclofenac	DCF	4.14	4.51	6.14 × 10^−8^	296.16	C_14_H_10_C_l2_NO_2_
Ibuprofen	IBF	4.91	3.97	1.162 × 10^−11^	206.23	C_13_H_18_O_2_
Triclosan	TCS	7.9	4.76	4.0 × 10^−6^	444.44	C_22_H_24_N_2_O_8_

**Table 2:** Water quality in the study area.

Item	Value
pH	6.8 ± 0.2
Turbidity (NTU)	0.54 ± 0.34
TDS (mg/L)	325 ± 105
TOC (mg/L)	5.2 ± 1.7
UV_254_ (cm^−1^)	0.088 ± 0.020
BOD_5_ (mg/L)	1.8 ± 0.5
T-N (mg/L)	14.12 ± 3.40
T-P (mg/L)	0.169 ± 0.080

**Table 3:** Summary of MS/MS conditions for the target compound analysis.

Compounds	Precursor Ion (m/z)	Product Ion (m/z)	RT (min)	Collision Energy (eV)
Acetaminophen	152 [M + H]^+^	65	2.28	10
Atenolol	267 [M + H]^+^	116	2.04	30
Ranitidine	315 [M + H]^+^	176	2.05	30
Paraxanthine	180 [M + H]^+^	149	2.58	24
Iopromide	791 [M + H]^+^	774	2.45	50
Caffeine	195 [M + H]^+^	83	3.17	20
Sulfamethazine	279 [M + H]^+^	124	3.22	30
Trimethoprim	291 [M + H]^+^	123	2.87	20
Sulfamethoxazole	254 [M + H]^+^	92	3.61	10
Lincomycin	407 [M + H]^+^	126	2.89	30
Propranolol	206 [M + H]^+^	116	5.19	20
Carbamazepine	237 [M + H]^+^	179	6.31	20
Naproxen	231 [M − H]^−^	141	7.41	10
Diclofenac	296 [M − H]^−^	213	8.49	5
Ibuprofen	205 [M − H]^−^	161	8.66	20
Triclosan	288 [M − H]^−^	35	2.87	20
Acetaminophen-d_4_	156 [M + H]^+^	114	2.34	10
Atenolol-d_7_	247 [M + H]^+^	145	2.07	30
Ranitidine-d_6_	343 [M + H]^+^	207	2.03	20
Paraxanthine-d_3_	183 [M + H]^+^	124	2.52	30
Iopromide-d_3_	792 [M + H]^+^	603	2.32	50
Caffeine-d_9_	207 [M + H]^+^	150	3.12	20
Trimethoprim-d_9_	300 [M − H]^+^	123	2.83	10
Sulfamethoxazole-d_4_	259 [M − H]^+^	97	3.57	10
Lincomycin-d_3_	408 [M − H]^+^	126	2.88	30
Propranolol-d_7_	268 [M + H]^+^	116	5.17	20
Carbamazepine-d_10_	247 [M − H]^+^	201	6.24	30
Naproxen-d_4_	233 [M − H]^−^	189	7.47	10
Diclofenac-d_4_	298 [M − H]^−^	254	8.42	10
Ibuprofen-d_3_	208 [M − H]^−^	164	8.59	5
Triclosan-d_3_	290 [M − H]^−^	35	2.88	20
Sulfamethazine-^13^C_6_	162 [M − H]^+^	98	3.58	30

**Table 4:** Linearity, limits of method detection (MDLs), and quantification (MQLs) of the LC-MS/MS method.

Compounds	IS	Linear Range (μg/L)	MDL (ng/L)	MQL (ng/L)	RSD (%) (*n* = 5)	Accuracy (%)
Acetaminophen	Acetaminophen-d_4_	0.01–100	2.8	8.9	7.4	85.3
Atenolol	Atenolol-d_7_	0.05–100	5.6	20.7	11.2	85.5
Ranitidine	Ranitidine-d_6_	0.05–100	7.8	25.5	4.4	87.7
Paraxanthine	Paraxanthine-d_3_	0.5–100	103.4	328.9	3.3	99.5
Iopromide	Iopromide-d_3_	1–100	4.5	59.4	5.7	95.8
Caffeine	Caffeine-d_9_	0.05–100	7.2	24.1	9.3	86.3
Sulfamethazine	Sulfamethazine-^13^C_6_	0.01–100	4.7	3.8	8.9	95.4
Trimethoprim	Trimethoprim-d_9_	0.05–100	3.4	11.4	15.4	95.7
Sulfamethoxazole	Sulfamethoxazole-d_4_	0.01–100	3.4	3.6	5.0	104.9
Lincomycin	Lincomycin-d_3_	0.02–100	4.4	11.4	9.4	85.8
Propranolol	Propranolol-d_7_	0.01–100	3.4	3.6	12.0	92.9
Carbamazepine	Carbamazepine-d_10_	0.05–100	3.4	11.4	8.1	88.2
Naproxen	Naproxen-d_3_	0.02–100	2.5	8.4	6.5	100.6
Diclofenac	Diclofenac-d_4_	0.02–100	2.5	8.4	8.5	95.1
Ibuprofen	Ibuprofen-d_3_	0.1–100	16.2	54.0	6.5	97.6
Triclosan	Triclosan-d_3_	0.05–100	7.8	25.9	5.4	86.4

**Table 5:** Matrix effect of 16 PPCPs.

Compounds	Matrix Effect					
	1 (μg/L)		5 (μg/L)		20 (μg/L)	
	Mean (%)	RSD (%)	Mean (%)	RSD (%)	Mean (%)	RSD (%)
Acetaminophen	82.4	9.2	84.9	7.4	103.5	5.2
Atenolol	103.1	14.1	106.3	12.1	112.7	9.8
Ranitidine	87.2	9.2	89.9	9.4	114.6	5.4
Paraxanthine	69.5	5.4	73.2	3.4	87.5	3.4
Iopromide	110.7	6.7	114.1	5.7	88.6	5
Caffeine	109.5	6.36	112.9	5.6	112.8	5.6
Sulfamethazine	89.3	6.7	92.1	6	113.8	5.8
Trimethoprim	97.4	5.5	104.4	4.5	112.9	3.5
Sulfamethoxazole	75	8.9	78.9	5.6	84.7	5.1
Lincomycin	85.9	7.5	86.5	6.9	104.6	5.3
Propranolol	132.6	6.4	136.5	5.4	113.7	4.2
Carbamazepine	92.1	6.9	95.1	6.2	110.5	3.2
Naproxen	93.7	6.8	96.6	6.5	102.8	3.2
Diclofenac	121.3	8.3	120.7	6.3	112.3	3.3
Ibuprofen	71.8	10.4	75.6	8.4	80.4	6.9
Triclosan	58.8	8.9	61.9	5.4	97.9	6.5

## Data Availability

Data are available on request from the corresponding author.
